# Supplementary material for: Loneliness and the persistence of fear: Perceived social isolation reduces evaluative fear extinction
Source: PLoS One. 2024 Aug 19;19(8):e0303895. doi: 10.1371/journal.pone.0303895 (PMC11333009; doi:10.1371/journal.pone.0303895)
Supplement: S1 File — (DOCX) [file pone.0303895.s001.docx]

Loneliness and the persistence of fear: Perceived social isolation reduces evaluative fear extinction

Erica Hornstein, Lee Lazar, & Naomi Eisenberger

Supplementary Information

Results

Evaluative Acquisition: Examined Using Only Participants Who Completed Days 1 & 14

While examination of evaluative acquisition (using liking scores) is reported for all participants who completed acquisition procedures on Day 1, some of these participants did not return for Day 14 to complete the extinction procedures. Therefore, here we provide evaluative acquisition results for *only participants who completed both Days 1 and 14.*

We found that a comparison of evaluative responding (liking ratings) for each condition (positive-paired, neutral-paired, fear-paired) across low vs. high lonely groups revealed no interaction (F(1.701,1326.640) = .621, *p = .512*, η^2^ = .001: Greenhouse-Geisser reported as Mauchly’s test had a *p < .01*), and only a main effect of pairing condition (F(1.701,1326.640) = 80.660, *p < .001*, η^2^ = .094: Greenhouse-Geisser reported as Mauchly’s test had a *p < .01*), such that positive-paired target images were rated more highly positive than either neutral-paired (*p < .001*, 95% CI [.188 .333]) or fearful-paired (*p < .001*, 95% CI [.352, .557]) target images, and neutral-paired target images were more highly rated than fear-paired target images (*p < .001*, 95% CI [.113, .275]: Bonferonni corrections applied on all pairwise comparisons). *Thus, the results for only participants who completed Days 1 and 14 mirror the results found for all of the participants who completed Day 1*, showing that evaluative appetitive responses were acquired in the positive-paired condition, indicated by higher positivity ratings compared the neutral-paired condition, and evaluative fear responses were acquired in the fearful-paired condition, indicated by lower positivity ratings compared to the neutral-paired condition.

Evaluative Learning: Examined Using Affective Ratings

*Evaluative Acquisition.* Using affective ratings, we found that a comparison of evaluative responding (positivity/negativity ratings) for each condition (positive-paired, neutral-paired, fear-paired) across low vs. high lonely groups revealed no interaction (F(1.642,1647.013) = .787, *p = .433*, η^2^ = .001: Greenhouse-Geisser reported as Mauchly’s test had a *p < .01*), and only a main effect of pairing condition (F(1.642,1647.013) = 120.915, *p < .001*, η^2^ = .108: Greenhouse-Geisser reported as Mauchly’s test had a *p < .01*), such that positive-paired target images were rated more highly positive than either neutral-paired (*p < .001*, 95% CI [.236, .373]) or fearful-paired (*p < .001*, 95% CI [.434, .634]) target images, and neutral-paired target images were more highly rated than fear-paired target images (*p < .001*, 95% CI [.152, .306]: Bonferonni corrections applied on all pairwise comparisons). *Thus, the results for the affective ratings during evaluative acquisition mirror the results found for liking ratings*, showing that evaluative appetitive responses were acquired in the positive-paired condition, indicated by higher positivity ratings compared the neutral-paired condition, and evaluative fear responses were acquired in the fearful-paired condition, indicated by lower positivity ratings compared to the neutral-paired condition.

However, when comparing evaluative responding using affective ratings during evaluative extinction, we found that a comparison of evaluative responding (positivity/negativity ratings) for each condition across low (n = 308) and high ( n = 474; these numbers reflect participants who completed both day 1 & day 14) lonely groups during acquisition revealed an overall not significant interaction of loneliness (high, low) x procedure (acquisition, extinction) x condition (positive-paired, neutral-paired, fearful-paired) (*p = .453 with intervention covariate; .427 without intervention covariate*).

These results did show a main effect of condition, just as found with the liking scores, (F(1.843, 1435.466) = 12.994, *p < .001*, η^2^ = .016 *with intervention covariate*; F(1.843, 1437.303) = 87.943, *p < .001*, η^2^ = .101 *without intervention covariate*: Greenhouse-Geisser reported as Mauchly’s test had a *p < .01*), such that positive-paired target images were rated more liked than either neutral-paired (*p < .001,* 95% CI [.139, .254] *with/without intervention covariate*) or fear-paired target images (*p < .001*, 95% CI [.276, .419] *with/without intervention covariate*), and neutral-paired target images were rated more liked than fearful-paired target images (*p < .001,* 95% CI [.092, .210] *with/without intervention covariate*: for all, Bonferonni corrections applied on all comparisons), mirroring the liking ratings and showing that the learning procedure was successful. Furthermore, just as with the liking ratings, there was a main effect of loneliness (F(1, 779) = 7.507, *p = .006*, η^2^ = .010 *with intervention covariate*; F(1, 780) = 7.336, *p = .007*, η^2^ = .009 *with intervention covariate*), such that high lonely individuals exhibited overall lower liking ratings compared to low lonely individuals (*p = .006*, 95% CI [.041, .247] *with intervention covariate*; *p = .007*, 95% CI [.039, .245] *without intervention covariate*: for all, Bonferonni correction applied).

Evaluative Extinction: Examined Without Intervention Covariate (Liking Scores)

In the study we provide the results of an examination of evaluative extinction (liking scores) using the intervention from the larger study as a covariate to account for any effects this intervention may have had on results. However, when we look at the results without intervention as a covariate, the pattern of effects does not change. In particular, the comparison of evaluative responding (liking ratings) for each condition across low (n = 308) and high ( n = 474) lonely groups during acquisition (directly following acquisition procedures) and extinction (two-week follow-up post extinction procedures) revealed an overall significant interaction of loneliness (high, low) x procedure (acquisition, extinction) x condition (positive-paired, neutral-paired, fearful-paired) (F(1.853,1444.381) = 3.467, *p = .035*, η^2^ = .004: Greenhouse-Geisser reported for Mauchly’s test had a *p < .01*). Further investigation revealed the same patterns of appetitive extinction occurred in both low and high lonely individuals compared to when intervention was included as a covariate, indicated by decreased liking for positive-paired images from acquisition to extinction (low lonely: t(307) = 3.829,  *p < .001*, 95% CI [.054, .10]; high lonely: t(473) = 6.089, *p < .001*, 95% CI [.18, .35]: Bonferonni corrections applied on all comparisons). In the neutral-paired condition, there was no change in responding from acquisition to extinction in the low lonely group (*p = .246*), reflecting the lack of acquired evaluative response to be reduced, but there was a decrease in liking in the high lonely group (t(473) = 2.575, *p = .01*, 95% CI [.04, .03]: Bonferonni correction applied). In the fearful-paired condition, extinction of evaluative fear responses occurred only in the low lonely condition, indicated by increased liking from acquisition to extinction (t(307) = -4.006, *p < .001*, 95% CI [-.38, -.13]: Bonferonni correction applied). However, no such extinction occurred for high lonely individuals in the fearful-paired condition (change from acquisition to extinction was not significant: *p = .689*).

Analyses further revealed a main effect of condition (F(1.867,1456.408) = 54.172, *p < .001*, η^2^ = .065: Greenhouse-Geisser reported as Mauchly’s test had a *p < .01*), such that positive-paired target images were rated more liked than either neutral-paired (*p < .001,* 95% CI [.129, .243]) or fear-paired target images (*p < .001*, 95% CI [.196, .338]), and neutral-paired target images were rated more liked than fearful-paired target images (*p = .004,* 95% CI [.020, .141]: Bonferonni corrections applied on all comparisons), indicating that, as found in the acquisition data, the learning procedure was successful in training participants to associate positive, neutral, or fearful associations with target images and similar patterns existed post extinction. These results also revealed a main effect of loneliness (F(1, 779) = 7.251, *p = .007*, η^2^ = .009), such that high lonely individuals exhibited overall lower liking ratings compared to low lonely individuals (*p = .007*, 95% CI [.038, .244]: Bonferonni correction applied). All of these results mirror those found with the intervention covariate.

However, the removal of the intervention covariate did influence the main effect of procedure (acquisition, extinction), such that it became significant (F(1,780) = 4.803, *p = .029*, η^2^ = .006: Greenhouse-Geisser reported as Mauchly’s test had a *p < .01*), such that liking ratings overall decreased from acquisition to extinction (*p =.029*, 95% CI [.007, .119]. Although this effect is not very strong, it likely reflects the impact of the intervention on overall positivity over time.
