# Supplementary material for: Loneliness and the persistence of fear: Perceived social isolation reduces evaluative fear extinction
Source: PLoS One. 2024 Aug 19;19(8):e0303895. doi: 10.1371/journal.pone.0303895 (PMC11333009; doi:10.1371/journal.pone.0303895)
Supplement: Attachment — Submitted filename: Response to Reviewers.docx [file pone.0303895.s002.docx]

Dear Dr. Akaishi,

Thank you for giving us the opportunity to resubmit our manuscript along with changes based on your and the reviewer’s suggestions. We feel that the feedback we received has helped to make the findings reported more accessible and the manuscript stronger overall. Below, we have provided specific responses to the comments and concerns raised as well as the journal requirements.

**Comments from the Editor:**
**The manuscript has elicited positive feedback from both reviewers, who have acknowledged the commendable execution of the study and the articulation of the paper. Nonetheless, both reviewers have highlighted significant concerns regarding the manuscript's positioning within the extensive body of literature on loneliness and fear conditioning. A particular point of contention is the methodological approach adopted in the study, notably the preference for subjective reports over objective measures such as skin conductance response. This methodological choice has been identified as a potential weakness by one reviewer, a critique that finds some justification within the traditional animal literature context. However, it is also pertinent to note that recent discussions within the field have begun to challenge the validity of fear conditioning as a reliable measure of fear. The reliance on subjective reports for assessing both loneliness and fear conditioning/extinction introduces complex issues that merit careful consideration. We strongly encourage the authors to address these critical points, alongside the detailed feedback provided by the reviewers, to enhance the manuscript's contribution to the field.**

We appreciate this feedback and would to note that we have addressed reviewer concerns regarding the use of subjective concerns during the evaluative conditioning procedures (please see the response to *Reviewer 2, comment 4*). With regard to recent discussions regarding the value and validity of fear conditioning, it is important to note that while different conceptualizations of the emotion of fear have been presented in the last decade—fear conditioning, and the outcomes that result from it, has a long and studied history that is not only reliable in both animals and humans but has been demonstrated to translate to both pathological processes as well as their treatments. These important and documented contributions of fear conditioning as a measure of fears and dysfunctional fears—as well as how to mediate these experiences—renders the continued study of fear conditioning an extremely important contribution to the field.

**Comments to the Author**

**1. Is the manuscript technically sound, and do the data support the conclusions?

The manuscript must describe a technically sound piece of scientific research with data that supports the conclusions. Experiments must have been conducted rigorously, with appropriate controls, replication, and sample sizes. The conclusions must be drawn appropriately based on the data presented.**

**Reviewer #1: Yes**

**Reviewer #2: Yes**

**2. Has the statistical analysis been performed appropriately and rigorously?**

**Reviewer #1: Yes**

**Reviewer #2: Yes**

**3. Have the authors made all data underlying the findings in their manuscript fully available?

The** [**PLOS Data policy**](http://www.plosone.org/static/policies.action#sharing) **requires authors to make all data underlying the findings described in their manuscript fully available without restriction, with rare exception (please refer to the Data Availability Statement in the manuscript PDF file). The data should be provided as part of the manuscript or its supporting information, or deposited to a public repository. For example, in addition to summary statistics, the data points behind means, medians and variance measures should be available. If there are restrictions on publicly sharing data—e.g. participant privacy or use of data from a third party—those must be specified.**

**Reviewer #1: No**

As noted, we were waiting until all papers stemming from this dataset were both in the publication process to make this data publicly available. However, regardless of their status, upon acceptance of this work we will make the data publicly available on the Harvard Dataverse.

**Reviewer #2: Yes**

**4. Is the manuscript presented in an intelligible fashion and written in standard English?

PLOS ONE does not copyedit accepted manuscripts, so the language in submitted articles must be clear, correct, and unambiguous. Any typographical or grammatical errors should be corrected at revision, so please note any specific errors here.**

**Reviewer #1: Yes**

**Reviewer #2: Yes**

**Reviewer #1:**

**This manuscript presents a large-scale study of the effects of loneliness on the extinction of evaluative fear learning. The authors extend with this work their previous findings with classical fear conditioning. The topic of the manuscript is of interest and importance to better understand how loneliness may have an impact on fear extinction processes and therefore also clinical fear exposure. The methods are sound and the manuscript is well written.**

Thank you so much for this kind feedback. We very much appreciate your suggestions and have responded to each below.

**I just have a few minor points that I would like to see implemented:**

**1. Abstract: Please add more information about the methods used. Given the large sample size used, it would also be good to see the final n used for the extinction analysis already here.**

We have included more description of the evaluative conditioning methods used in the study as well as the final sample size used to assess the strength of fear conditioning in our analyses (please see lines 43-48).

**2. Throughout the manuscript there are frequent references to manuscripts in preparation or submitted. I am not sure what Plos One's policy is on this, but I would prefer to see a reference to a pre-registration or preprint so that the reader can actually read the source of the reference. For example, in the Methods "(see: L. Lazar, EAH (author), & 181 NIE (author), in prep))**

Unfortunately, these papers are in prep and were not pre-registered/do not yet have pre-prints to reference at this point. We are happy to remove these citations if the journal requests us to do so, however we do think they are important for the paper itself—especially the reference noted above which refers to the other paper that will be published based on the data collected during this study. We have updated this reference, as this other paper is closer to being submitted, and it is our hope that by the time of the publication of this manuscript, the other paper stemming from this data will be further along in the publication process and we can refer instead to a preprint.

**3. Methods: "(...) protocols designed to examine the effects of a prosocial behavior intervention on feelings of well-being" There is no indication that the study was pre-registered. Was this analysis or the larger study pre-registered somewhere? If so, please provide a reference.**

Unfortunately, although the hypotheses for this study were spelled out in the grant that funded it, neither this study nor the analyses used here were officially pre-registered so we cannot reference this in the current manuscript.

**4. It’s not clear what is used as the “9 target neutral images”. Are these also images from the IAPS with the same neutral content as the secondary neutral images used for the evaluative conditioning? Please clarify this in the methods.**

We have now added more detail regarding the 9 target neutral images (which were all images of everyday objects on a white background). Please see lines 238-240 in the revised manuscript.

**5. l.364, l.393 The justification of the results should be in the Discussion section only.**

We have removed the justification language from line 364 (now line 399), but were unable to determine which language the reviewer found inappropriate on line 393. We would be happy to make further adjustments with further clarification.

**Reviewer #2:**

**This paper studied the fear learning and extinction in high and low loneliness participants. And the authors found that the patterns of the extinction of the evaluative fear associations were different between the high and low individuals. This manuscript presents a compelling and well-executed study that significantly contributes to the field of social bonds on fear learning. The authors demonstrate a thorough understanding of the research topic and employ a rigorous methodology to investigate their research questions. Overall, the manuscript is well-written, engaging, and likely to generate interest among readers.**

We appreciate these encouraging words and that the reviewer feels this work represents a contribution to the field. We have addressed the reviewer’s suggestions to improve the paper below.

**However, there are still some questions in the manuscript. The details are as follows:**

**Major questions
1. In the Introduction, the authors mentioned the negative influence of perception of being socially isolated on mental health outcomes and they also mentioned that no study have invested the associations between loneliness and fear learning. The definition between perception of social isolation and social exclusion are very similar. According to previous study (Cacioppo&Cacioppo, 2016), Social exclusion has been defined broadly as “the experience of being kept apart from others physically or emotionally， Therefore, perception of social isolation, ostracism, and romantic rejection fall under the broad rubric of social exclusion”. Thus, Dou et al.(2020) have already investigated that the influences of social exclusion on fear learning in humans and found the participants after social exclusion inhibited fear conditioning compared with those after social inclusion. Surprisingly, I found the authors introduced lots of animal’s studies, rather than mentioned this closely related paper in humans in the introduction section.**

The paper the reviewer points out is such an excellent paper and a very important contribution to the growing understanding of the role of social connection or disconnection in fear learning. In the case of subjective social isolation (loneliness) or objective social isolation, it is important to note that these experiences are defined by either a perceived lack of close relationships or an actual lack of relationships/contact that is chronic and globally experienced across an individual’s life—not by the acute and intentional removal of a relationship that is experienced in social exclusion. For this reason, we felt that these experiences were too dissimilar to compare across, even though they share similarities (just as it is difficult to compare across the effects of chronic and acute pain). However, it is clear that these studies all fall under the same umbrella of building knowledge of the powerful role of social connection on fear learning and therefore we have included discussion of this work in the introduction of our revised manuscript (please see lines 131-135).

**2. In the introduction and discussion sections, the authors used a large amount sentence to interpret that the anxiety may play an important role in relation between loneliness and fear extinction. However, the author did not report any anxiety score for each participant. As the authors mentioned, the individuals with anxiety disorders showed a dysfunction patterns during the classical conditioning and extinction (Lissek et al., 2005; Duits et al., 2015). Whether the high loneliness groups showed higher anxiety scores compared to the low loneliness groups? Thus, whether the different patterns of extinction in high/low loneliness were also due to the differences of anxiety scores?**

The reviewers raise an important question here and in their next comment. We did collect anxiety and depression reports from participants (using the State/Trait Anxiety Scale and the Beck Depression Inventory, respectively) and examination of these scores revealed that, when split into low vs high groups (like those used to assess loneliness), neither anxiety nor depression showed any significant interaction with the change from acquisition to extinction in any condition (positive, neutral, or fearful), suggesting that neither of these experiences affect fear extinction in the way that loneliness was found to have done. Further, correlation analyses showed that neither anxiety nor depression had any significant relationship with change of ratings from acquisition to extinction, once again showing these scores don’t appear to influence fear extinction in the same way loneliness did in our results.

**3. Similarly, the lots of previous studies(Cacioppo et al., 2006; Erzen & Çikrikci, 2018; Matthews et al., 2016; Singh & Misra, 2009) have already reported loneliness showed a close relationship with depression, even in different ages, such as youth, adult, and elderly. Moreover, previous research have reported individuals with depression showed dysfunction in affective processing, especially the reward learning(Admon & Pizzagalli, 2015). During this study, whether the depression showed a significant difference between the high and low loneliness group? And what role did the depression play between the loneliness and reward learning and extinction?**

As expected, we did find a significant difference in depression scores across the high and low lonely groups (p = .041), yet we found no effect of depression on fear extinction outcomes (please see response to above comment: Reviewer 2, comment 2). Interestingly, we did not find a significant difference in anxiety scores across the low and high lonely groups (p > .1). As with depression scores, we found no effect of anxiety on fear extinction outcomes (again, please see response to above comment: Reviewer 2, comment 2).

**4. In the previous fear learning and extinction paradigm, compared to subjective ratings, some physiological responses to conditioned stimulus(CS) (such as skin conductance responses, SCR) were more widely used((Lonsdorf et al., 2017). Thus, the dependent variables in this manuscript were only the subjective ratings, which might influence accuracy of the results of the acquisition and extinction. I think this is a limitation in this manuscript.**

We appreciate the reviewer raising this issue. While subjective ratings and measurements of physiological responding are certainly different in nature, there is evidence regarding not only the value of subjective reporting but also the connection between this type of reporting/evaluative conditioning and the physiological measures often used in in-lab Pavlovian conditioning procedures. We have therefore added discussion of these important issues in the revised manuscript (see lines 170-179). However, we agree with the reviewer that evaluative conditioning on its own is not the same as in-person Pavlovian procedures that are often used to assess fear learning. Therefore, we have addressed this limitation and the need for future work to investigate the relationship between loneliness and non-evaluative, Pavlovian fear conditioning using non-reported, physiological measures in the discussion (please see lines 546-554 in the revised manuscript).

**5. Similarly, the habitation stage was also common adopted in the conditioning studies, which could reduce the extra variable evoked by the stimuli. However, the authors did not adopt the habitation stage in this study.**

The reviewer raises an important question regarding the use of the habituation stage Indeed, the use of habituation for CSs is common in in-person conditioning procedures to ensure that participants are familiar with the conditional stimuli so that they no longer elicit physiological responding due to surprise at their appearing and/or their novelty. This also allows for examination of any pre-existing characteristics of any of the stimuli that continue to elicit differential responding even after familiarity is achieved. However, habituation is not always used and indeed can have drawbacks, including visual and attentional fatigue. Additionally, in cases when subjective reports, not physiology, are being measured, the need to reduce responding due to novelty is not necessary—for while novelty or lack of experience with images occurring on the screen might lead to increases in sympathetic nervous system responding, it is unlikely to lead to differential increases in emotional responding or liking. Finally, with regard to pre-existing characteristics of stimuli that might lead to differential responding that is not due to learning, the use of equally familiar (everyday objects) content that is presented in the same way (object on white background) is likely to ensure to that none of the objects portrayed for the CSs were emotionally charged. Importantly, to further address any potential pre-existing characteristics, the pairings of CSs with different types of USs (positive, fearful, neutral) were counterbalanced across participants (see lines 251-253), ensuring that these pre-existing characteristics would be equally applied across conditions. For these reasons, we did not include habituation in the current study.

**6. Although the influence of the different interventions (including prosocial behavior intervention, self-kindness internation, and control) are calculated, the influence of on the extinction might also mixed in the experiment, which might be better to add in the limitation section.**

We have now added discussion of this potential limitation to the discussion section of the revised manuscript (please see lines 546-554).

**7. Although the authors set the cut-off for the loneliness or not, I am still curious about the correlation between UCLA scores and the ratings in both of the acquisition and extinction throughout the whole samples.**

The reviewer raises an interesting question and we did look at the correlation between scores on the UCLA v3 change in ratings from acquisition to extinction (reflecting extinction) across the sample, but while some of these correlations achieved significant p values (change in affect ratings for fear reinforced cues (p=.002); change in liking ratings for fear reinforced cues (p=.013) & positive reinforced cues (p=.034)) and trended in the same direction as the reported results (with higher loneliness leading to less extinction of fear associations), the R^2^ values were low (all R^2^s ≤ .008) suggesting that these correlations were not very strong and should not be interpreted as meaningful. This may be due to the fact that while a large sample was recruited for this study, participants were not specifically recruited based on their loneliness status. Therefore, while we do have a large amount of high lonely individuals in the study, including many who are extremely lonely, there are still more participants who are in the middle range of the high lonely group than the highest range, leading to a weaker linear trend (see example chart for the correlation between loneliness score and change in liking scores from acquisition to extinction). Regardless, it is notable that these correlations were significant, even if the R^2^s remained low, suggesting that the trend of higher loneliness leading to poorer extinction is a good fit for this relationship.

**Minor questions
1. The figure of the procedure was not clear enough. What were the details in the acquisition section? Whether the CS and US presented on the screen at the same time or not?**

We have added “next screen” under the arrows in the figure in order clarify that the CS and US were not presented on the same screen, but consecutively. Furthermore, we have clarified this in the caption for figure 1 (please see lines 258-259 in the revised manuscript).

**2. In figures of acquisition and extinction, the error bar were missing, which could be added to improve the quality of the figures.**

Thank you for pointing this out--we apologize for this oversight and have now added the error bars to these figures.

**Journal Requirements**

**1. Please ensure that your manuscript meets PLOS ONE's style requirements, including those for file naming.**

We have updated the manuscript to meet the PLOS ONE style requirements and have adjusted our file names as well.

**2. Thank you for stating the following in the Acknowledgments Section of your manuscript:**

**"This work was funded by a National Science Foundation RAPID Grant (NSF 2034809) awarded to E.A.H. & N.I.E."**

**We note that you have provided additional information within the Acknowledgements Section that is not currently declared in your Funding Statement. Please note that funding information should not appear in the Acknowledgments section or other areas of your manuscript. We will only publish funding information present in the Funding Statement section of the online submission form.**

**Please remove any funding-related text from the manuscript and let us know how you would like to update your Funding Statement. Currently, your Funding Statement reads as follows:**

**"National Science Foundation RAPID Grant award to to N.I.E. and E.A.H (NSF 2034809:** [**https://www.nsf.gov**](https://www.nsf.gov)**). The sponsors played no role in study design, data collection/analysis, decision to publish, or preparation of the manuscript. "**

**Please include your amended statements within your cover letter; we will change the online submission form on your behalf.**

We apologize for including this information twice and have removed this language from the acknowledgements section of our manuscript and have confirmed that the information in the Funding Statement is correct.

**3. Please include your full ethics statement in the ‘Methods’ section of your manuscript file. In your statement, please include the full name of the IRB or ethics committee who approved or waived your study, as well as whether or not you obtained informed written or verbal consent. If consent was waived for your study, please include this information in your statement as well.**

We have included the requested information in the ethics statement section of our manuscript, including information on how informed consent was received. Please see lines 202-206 in the revised manuscript.

**4. In this instance it seems there may be acceptable restrictions in place that prevent the public sharing of your minimal data. However, in line with our goal of ensuring long-term data availability to all interested researchers, PLOS’ Data Policy states that authors cannot be the sole named individuals responsible for ensuring data access (**[**http://journals.plos.org/plosone/s/data-availability#loc-acceptable-data-sharing-methods**](http://journals.plos.org/plosone/s/data-availability#loc-acceptable-data-sharing-methods)**).**

**Data requests to a non-author institutional point of contact, such as a data access or ethics committee, helps guarantee long term stability and availability of data. Providing interested researchers with a durable point of contact ensures data will be accessible even if an author changes email addresses, institutions, or becomes unavailable to answer requests.**

**Before we proceed with your manuscript, please also provide non-author contact information (phone/email/hyperlink) for a data access committee, ethics committee, or other institutional body to which data requests may be sent. If no institutional body is available to respond to requests for your minimal data, please consider if there any institutional representatives who did not collaborate in the study, and are not listed as authors on the manuscript, who would be able to hold the data and respond to external requests for data access? If so, please provide their contact information (i.e., email address). Please also provide details on how you will ensure persistent or long-term data storage and availability.**

As noted above, we are waiting for the other paper using data from this data set to begin the publication process before making the data publicly available. However, regardless of the other manuscript’s status, when this paper is going into press we will make the data publicly available on the Harvard Dataverse. For this reason, we have not provided non-author contact information for a data access committee, as the data will be publicly available upon the date of publication. Please let us know if this solution works.

**5. PLOS requires an ORCID iD for the corresponding author in Editorial Manager on papers submitted after December 6th, 2016. Please ensure that you have an ORCID iD and that it is validated in Editorial Manager. To do this, go to ‘Update my Information’ (in the upper left-hand corner of the main menu), and click on the Fetch/Validate link next to the ORCID field. This will take you to the ORCID site and allow you to create a new iD or authenticate a pre-existing iD in Editorial Manager. Please see the following video for instructions on linking an ORCID iD to your Editorial Manager account:** [**https://www.youtube.com/watch?v=_xcclfuvtxQ**](https://www.youtube.com/watch?v=_xcclfuvtxQ)**.**

We have updated the corresponding authors account in the editorial manager so that it is linked with her ORCID ID. Please let us know if there is any further issue.

**6. Please review your reference list to ensure that it is complete and correct. If you have cited papers that have been retracted, please include the rationale for doing so in the manuscript text, or remove these references and replace them with relevant current references. Any changes to the reference list should be mentioned in the rebuttal letter that accompanies your revised manuscript. If you need to cite a retracted article, indicate the article’s retracted status in the References list and also include a citation and full reference for the retraction notice.**

We have reviewed our reference list and don’t believe we have cited any retracted papers. However, we have cited two papers that are in prep, one of which is also based on this data set. We are not certain of the journal’s policy on work that is in prep (and it is our hope that the other paper based on this data set will be further along in the publication process by the time this paper is in press), so we are willing to remove these references if it is required.
